# Supplementary material for: Anisotropic ESCRT-III architecture governs helical membrane tube formation
Source: Nat Commun. 2020 May 29;11:1516. doi: 10.1038/s41467-020-15327-4 (PMC7260168; doi:10.1038/s41467-020-15327-4)
Supplement: Supplementary file 4 — Description of Additional Supplementary Files [file 41467_2020_15327_MOESM4_ESM.pdf]

Title: Supplementary Movie 1
Description: Scanning through slices of tomogram and rotation of filtered and segmented tomographic volumes as shown in Fig. 1c-e.

Title: Supplementary Movie 2
Description: Scanning through slices of tomogram and rotation of filtered and segmented tomographic volumes as shown in Supplementary Fig. 1 i-k.
